# Association of the interaction between nutritional status and frailty level with sleep quality in older adults

**DOI:** 10.3389/fpubh.2025.1615643

**Published:** 2025-09-23

**Authors:** Shaoting Yang, Yumeng Zhang, Zhixia Jiang, Heting Liang, Qingyun Mao, Honghong Wen, Yan Xiong, Yunting Li, Xiaoli Yuan

**Affiliations:** ^1^Department of Nursing, Affiliated Hospital of Zunyi Medical University, Zunyi, Guizhou, China; ^2^Faculty of Nursing, Zunyi Medical University, Zunyi, Guizhou, China; ^3^College Office, Guizhou Nursing Vocational College, Guiyang, Guizhou, China

**Keywords:** sleep quality, nutritional status, degree of frailty, interaction effect, older adults

## Abstract

**Background:**

Sleep quality decline is common among older adults, affecting their physical and mental health and reducing quality of life. Research shows a notable link between older adults’ nutritional status, frailty, and sleep quality.

**Objective:**

This study delves into the interaction between older adults’ nutritional status and frailty on sleep quality, aiming to offer scientific strategies for improving sleep.

**Methods:**

Via convenience sampling, a one-year cross-sectional survey was conducted on 14,021 seniors aged 60+ from 7 medical facilities, 3 communities, and 5 nursing homes in Zunyi, Guizhou. The survey covered general data, FRAIL Scale, MNA-SF, and PSQI. Logistic regression explored relationships, and a generalized linear model evaluated interactions.

**Results:**

Of the 14,021 older adults, 24.3% had poor sleep. Factors like gender, age, education, residence, income, habits, social engagement, physicals, exercise, and support was significantly associated with sleep quality (all *p* < 0.05). Logistic regression affirmed nutritional status (OR = 1.076–1.193) and frailty (OR = 3.472–6.050). After controlling variables, generalized linear model showed nutritional status (*B* = 0.007), frailty (*B* = 1.145), and their interaction (*B* = 0.214), being significant (*p* < 0.05).

**Conclusion:**

Malnutrition and frailty synergistically exacerbate sleep disturbances in older adults. Integrated interventions targeting nutritional support and frailty mitigation are critical to improving sleep health in this vulnerable population.

## 1 Introduction

Statistics show that by 2023, China had 296.97 million people aged 60 and above, constituting 21.1% of the total population (1). China’s aging trend indicates a long-term high-level aging society with a growing older adult population, making health issues a top priority (2). Sleep disorders, common in older adults (30–48% prevalence), involve sleep – wake dysfunctions. Chronic insomnia and poor sleep can cause social and cognitive decline, higher fall risks, and increased chronic disease incidence and mortality (3)^.^ They also raise the risk of cardiovascular diseases like coronary heart disease in older adults (4).

Malnutrition, resulting from insufficient intake or absorption (5), is more common in older adults due to age-related physical decline. Frailty, a syndrome of reduced physiological reserves and activity (6), is widespread. Around 68.6% of frail older adults face malnutrition risks (7), both negatively affecting their physical and mental health and quality of life.

Previous research (8, 9) reveals that in China, overweight/obese older adults may sleep better than normal-weight ones, while underweight older adults have poorer sleep. Frailty also directly impacts sleep quality, with frail patients sleeping worse than non-frail ones. However, few studies address the interaction between frailty and nutritional status on sleep quality. Thus, this study explored this interaction to improve older adults’ sleep and promote healthy aging.

## 2 Methods

### 2.1 Subjects

This study adopted a cross-sectional design and employed the convenience sampling method. From October 2022 to September 2023, a total of 14,021 older adult individuals aged 60 and above were recruited from 26 medical institutions, 6 communities, and 9 older adult care facilities in Zunyi City, Guizhou Province. Indicators for older adults were assessed using a face-to-face questionnaire. All respondents completed the survey on-site. The inclusion criteria for the research subjects were as follows: being 60 years old or above; having the capacity for normal communication; giving informed consent and voluntarily participating in the study. The exclusion criteria were: having a history of severe mental illness or severe cognitive impairment, rendering communication impossible; suffering from acute and critical conditions (such as shock, respiratory failure, acute heart failure, acute myocardial infarction, stroke, etc.) and thus being unable to cooperate with the survey; being in the acute exacerbation phase of chronic diseases or at the end-stage of diseases (with an expected survival time of less than 3 months). This study has been reviewed and approved by the Ethics Review Committee of the Affiliated Hospital of Zunyi Medical University (KLL2022-814).

### 2.2 Sample size

Decreased sleep quality in older adults is an important variable in this study. By referring to a large amount of literature, we know that the decline in sleep quality of older adults in China is about 33.8% (10). The minimum sample size of 8,596 was calculated according to the following formula. Based on the 95% confidence interval (CI), we calculated the minimum sample size of 8,596 using the following formula:


$$N=\frac{Z^{2}P\left(1-P\right)}{\delta^{2}}=\frac{1.96^{2}\times 0.338\left(1-0.338\right)}{0.01^{2}}=8596$$

### 2.3 Questionnaire

#### 2.3.1 Frailty status: the FRAIL scale

This scale was proposed in 2008 by geriatric experts of the International Association of Nutrition and Aging (IANA) based on the frailty phenotype and frailty index (11). The scale consists of the following five items: Here are the refined sentences to make the language more natural and academic: Fatigue: Have you experienced a feeling of fatigue during the majority of the past 4 weeks or throughout the entire four-week period? Increased resistance or decreased endurance: Do you encounter difficulties in ascending a single flight of stairs without pausing for a break midway or relying on the aid of walking assistive devices? Decline in Mobility: Do you find it challenging to walk a distance of 100 meters unassisted by any walking aids? Disease Condition: Are you currently suffering from five or more different diseases? Weight Loss: Has your body weight diminished by 5% or a greater percentage over the course of the past year? For each of the above items, answering “yes” is scored as 1 point, and answering “no” is scored as 0 point. The total score is 5 points. A score of 3 points or higher indicates frailty, a score of 1 to 2 points indicates pre-frailty, and a score of 0 point indicates no frailty. The overall Cronbach’s *α* coefficient of the scale is 0.826, indicating good reliability and validity (12).

#### 2.3.2 Nutritional status

In the present study, the Mini Nutritional Assessment-Short Form (MNA-SF) was employed as a screening tool for nutritional risk in older adults. As an internationally recognized, concise, and validated questionnaire, the MNA-SF is designed to rapidly identify the risk of malnutrition or malnutritional status in older adult individuals (13). It comprises 6 items, including weight loss in the past 3 months, body mass index (BMI; or calf circumference as an alternative), mobility, recent acute illness/psychological stress, neuropsychological issues (dementia/depression), and eating disorders. Scores range from 0 to 14, with results classified into three categories: 12–14 (good nutritional status), 8–11 (at risk of malnutrition), and 0–7 (malnourished). The MNA-SF is easy to administer, taking approximately 3–5 min, and is applicable to diverse settings such as communities, hospitals, and older adult care facilities (14).

#### 2.3.3 Sleep quality

The Pittsburgh Sleep Quality Index (PSQI), devised by Buysse, a psychiatrist from Pittsburgh, the U. S. (15)^,^ was employed to evaluate participants’ sleep quality over the past month. It is suitable for assessing the sleep quality of both patients with sleep and mental disorders and the general public. The PSQI consists of seven key components (16): subjective sleep quality, sleep latency, sleep duration, sleep efficiency, sleep disturbances, sleeping pill usage, and daytime functioning impact. Each component is scored on a 0–3 scale, with the total score ranging from 0 to 21. A lower total score indicates better sleep quality. The scale has a Cronbach’s *α* coefficient of 0.84, demonstrating good reliability and validity. In this study, In this study, the total sleep quality score ranged from 0 to 21, with >5 points indicating poor sleep quality.

#### 2.3.4 General Infor

Gender, age, education, residence type, living mode, monthly per-capita family income, alcohol and smoking statuses, annual-physical-exam participation, social-activity participation, exercise habits, social support, nutritional status, and sleep quality were chosen as control variables. Specific assignments are presented in Supplementary Table 1.

### 2.4 Statistical methods

Data were analyzed with SPSS 29.0. Enumeration data were presented as relative numbers. The *χ*^2^ test compared sleep quality among older adult groups with different demographic features. Logistic regression analyzed the links between older adults’ nutritional status, frailty degree, and sleep quality. Model 1 had no controlled variables; Model 2 controlled demographics and other confounders Confounding variables were controlled via professional judgment and statistics. Variables theoretically strongly correlated with outcomes were prioritized; additionally, in logistic regression analysis, when sleep quality was the dependent variable and nutritional status and frailty were the independent variables, a baseline model with only these variables was built. Individual addition of age, gender, etc., and a >10% change in sleep quality’s regression coefficient identified confounders: gender, age, education, residence type/arrangement, alcohol use, smoking, social activity participation, annual physical exams, and exercise.

Logistic models then included sleep quality (good = 1; poor = 2) as the dependent variable, and nutritional status (good = reference) and frailty (non-frail = reference) as independents (two models). A generalized linear model explored associations among nutritional status, frailty, their interaction, and sleep quality (all scores as continuous variables). Significance was *α* = 0.05.

## 3 Results

### 3.1 General information of the subjects

This study enrolled 14,021 older adult individuals aged 60 and above, of whom 7,100 (50.64%) were male and 6,921 (49.36%) were female, with a mean age of 71.0 years (*Q*₁ = 66.0, *Q*₃ = 76.0). In terms of residence, 7,157 (47.94%) lived in urban areas and 6,864 (52.06%) in rural areas. Regarding living arrangements, the majority (12,858, 92.09%) lived with family, 784 (5.61%) lived alone, and 321 (2.30%) were in nursing homes.

Study findings indicated that older adults’s sleep quality was significantly associated with gender, age, education level, residence type, living pattern, average monthly per capita family income, alcohol and smoking status, social activity participation, annual physical check-ups, exercise habits, and social support. All these correlations were statistically significant (*p* < 0.05), as presented in Table 1.

**Table 1:** Comparison of sleep quality differences among older adults with different demographic characteristics.

Variables	Total (*n* = 14,021)	Good sleep quality (*n* = 10,603)	Poor sleep quality (*n* = 3,418)	Statistic	*P*
Age, M (*Q*₁, *Q*₃)	71.00 (66.00–76.00)	70.00 (66.00–76.00)	72.00 (67.00–77.00)	*Z* = −9.78	<0.01
Gender				*χ*^2^ = 92.77	<0.01
Male	7,100 (50.64)	5,614 (52.95)	1,486 (43.48)		
Female	6,921 (49.36)	4,989 (47.05)	1932 (56.52)		
Educational level				*χ*^2^ = 22.43	<0.01
Below Junior High School and Junior High School	12,164 (86.94)	9,120 (86.18)	3,044 (89.32)		
Above Junior High School	1827 (13.06)	1,463 (13.82)	364 (10.68)		
Type of residence				*χ*^2^ = 71.76	<0.01
Urban	7,157 (51.04)	5,197 (49.01)	1960 (57.34)		
Rural	6,864 (48.96)	5,406 (50.99)	1,458 (42.66)		
Living pattern				*χ*^2^ = 52.91	<0.01
Living with family members	12,858 (92.09)	9,705 (91.89)	3,153 (92.71)		
Living alone	784 (5.61)	562 (5.32)	222 (6.53)		
Living in a nursing home	321 (2.30)	295 (2.79)	26 (0.76)		
Average monthly *Per Capita* family income				*χ*^2^ = 35.99	<0.01
≥2000 Yuan	7,201 (51.36)	5,598 (52.80)	1,603 (46.90)		
<2000 Yuan	6,820 (48.64)	5,005 (47.20)	1815 (53.10)		
Alcohol consumption status				*χ*^2^ = 29.09	<0.01
Non-drinker	12,209 (87.13)	9,139 (86.27)	3,070 (89.82)		
Drinker	1803 (12.87)	1,455 (13.73)	348 (10.18)		
Smoking status				*χ*^2^ = 8.85	0.003
Non-smoker	11,521 (82.40)	8,651 (81.86)	2,870 (84.09)		
Smoker	2,460 (17.60)	1917 (18.14)	543 (15.91)		
Participation in social activities				*χ*^2^ = 71.13	<0.01
Yes	8,828 (62.96)	6,883 (64.92)	1945 (56.90)		
No	5,193 (37.04)	3,720 (35.08)	1,473 (43.10)		
Annual physical examination				*χ*^2^ = 53.93	<0.01
Yes	7,098 (50.62)	5,181 (48.86)	1917 (56.09)		
No	6,923 (49.38)	5,422 (51.14)	1,501 (43.91)		
Physical exercise				*χ*^2^ = 14.07	<0.01
Yes	10,655 (75.99)	8,139 (76.76)	2,516 (73.61)		
No	3,366 (24.01)	2,464 (23.24)	902 (26.39)		
Social support status				*χ*^2^ = 30.45	<0.01
Sufficient	12,541 (89.44)	9,570 (90.26)	2,971 (86.92)		
Lack	1,480 (10.56)	1,033 (9.74)	447 (13.08)		
Nutritional status				*χ*^2^ = 7.36	0.025
Good nutritional status	4,773 (34.04)	3,612 (34.07)	1,161 (33.97)		
At Risk of malnutrition	7,816 (55.74)	5,868 (55.34)	1948 (56.99)		
Malnutrition	1,432 (10.21)	1,123 (10.59)	309 (9.04)		
Degree of frailty				*χ*^2^ = 1340.21	<0.01
No frailty	8,269 (58.98)	7,133 (67.27)	1,136 (33.24)		
Pre-frailty	3,794 (27.06)	2,445 (23.06)	1,439 (39.47)		
Frailty state	1958 (13.96)	1,025 (9.67)	933 (27.30)		

### 3.2 Influencing factors of older adults’ sleep quality

#### 3.2.1 Impact of older adults’ personal traits on sleep quality

Logistic regression model results indicated that, after accounting for demographic and confounding variables, various nutritional statuses of older adults (taking good nutritional status as the reference) exhibited a positive correlation with sleep quality (OR = 1.076–1.193). Conversely, different degrees of frailty in older adults (using no frailty as the reference) were positively associated with sleep quality (OR = 3.472–6.050), as shown in Table 2.

**Table 2:** Logistic regression analysis of the association between nutritional status, degree of frailty and sleep quality in older adults.

Independent variables	Options	Model 1		Model 2	
		OR (95% CI)	*P*	OR (95% CI)	*P*
Nutritional status (with good nutritional status as the reference)	At risk of malnutrition	1.099 (0.947–1.276)	0.214	1.076 (0.925–1.252)	0.34
	Malnutrition	1.210 (1.049–1.359)	0.009	1.193 (1.033–1.379)	0.017
Degree of frailty (with no frailty as the reference)	Pre-frailty	3.474 (3.170–3.806)	<0.001	3.472 (3.158–3.817)	<0.001
	Frailty State	5.734 (5.143–6.392)	<0.001	6.050 (5.384–6.799)	<0.001

#### 3.2.2 Multivariate analysis of sleep quality in older adults

Logistic regression analysis of older adults’ sleep quality: Taking poor sleep quality as the dependent variable and the variables with statistical significance in the univariate analysis as independent variables (assignments shown in Supplementary Table 1), the Logistic regression analysis was conducted. The results indicated that gender, age, educational level, type of residence, living pattern, average monthly per capita family income, alcohol consumption, smoking status, participation in social activities, annual physical examination, exercise, social support, nutritional status, and degree of frailty were all was significantly associated with, and the differences were statistically significant (*p* < 0.05), as shown in Table 3.

**Table 3:** Multivariate logistic regression analysis of influencing factors of sleep quality in older adults.

Variables	*β*	S. E	*Z*	*P*	OR (95%CI)
Age	0.026	0.003	9.610	<0.001	1.026 (1.021 ~ 1.032)
Gender (with male as the reference)					
Female	0.380	0.040	9.606	<0.001	1.463 (1.354 ~ 1.581)
Educational Level (with Below Junior High School and Junior High School as the reference)					
Above Junior High School	−0.294	0.062	−4.723	<0.001	0.745 (0.660 ~ 0.842)
Type of residence (with urban as the reference)					
Rural	−0.335	0.040	−8.453	<0.001	0.715 (0.662 ~ 0.773)
Living pattern (with living with family members as the reference)					
Living alone	0.195	0.082	2.387	0.017	1.216 (1.036 ~ 1.428)
Living in a nursing home	−1.305	0.205	−6.349	<0.001	0.271 (0.181 ~ 0.406)
Average monthly *Per Capita* family income (with ≥2000 Yuan as the reference)					
<2000 Yuan	0.236	0.039	5.993	<0.001	1.266 (1.172 ~ 1.368)
Alcohol consumption status (with non-drinker as the reference)					
Drinker	−0.340	0.063	−5.374	<0.001	0.712 (0.629 ~ 0.806)
Smoking status (with non-smoker as the reference)					
Smoker	−0.158	0.053	−2.972	0.003	0.854 (0.769 ~ 0.948)
Participation in social activities (with participation in social activities as the reference)					
No	0.337	0.040	8.416	<0.001	1.401 (1.295 ~ 1.516)
Annual physical examination (with annual physical examination as the reference)					
No	−0.290	0.040	−7.332	<0.001	0.748 (0.692 ~ 0.809)
Physical exercise (with physical exercise as the reference)					
No	0.169	0.045	3.748	<0.001	1.184 (1.084 ~ 1.294)
Social support status (with sufficient social support as the reference)					
Lack	0.332	0.060	5.499	<0.001	1.394 (1.238 ~ 1.569)
Nutritional status (with good nutritional status as the reference)					
At risk of malnutrition	0.032	0.043	0.756	0.450	1.033 (0.950 ~ 1.123)
Malnutrition	−0.155	0.073	−2.142	0.032	0.856 (0.743 ~ 0.987)
Degree of frailty (with no frailty as the reference)					
Pre-frailty	1.243	0.047	26.669	<0.001	3.464 (3.162 ~ 3.796)
Frailty state	1.743	0.055	31.472	<0.001	5.715 (5.127 ~ 6.371)

### 3.3 Association between the interaction effect of nutritional status and degree of frailty and sleep quality in older adults

Generalized linear model analysis showed in Model 1, which included the main effects of nutritional status score and frailty score and their interaction term, the frailty score was significantly positively was significantly associated with sleep quality (*B* = 1.146, *p* < 0.001), while the main effect of nutritional status was not significant (*B* = 0.011, *p* = 0.722). In Model 2, after further controlling for confounding factors such as gender, age, and educational level, the frailty score was still significantly was significantly associated with sleep quality (*B* = 1.145, *p* < 0.001), the interaction effect was significant (*B* = 0.214, *p* = 0.039), but the main effect of nutritional status still did not reach significance (*B* = 0.007, *p* = 0.926). These results indicated that the degree of frailty was an independent influencing factor of sleep quality, and it was further associated with the interaction of nutritional status, as shown in Table 4.

**Table 4:** Association between the interaction effect of nutritional status and degree of frailty and sleep quality in older adults.

Model	Variable	*B* (95% CI)	Standard error	Wald *X*^2^ value	*p*-value
Model 1	Nutritional status score	0.011(−0.127–0.149)	0.070	0.025	0.722
	Degree of frailty score	1.146 (0.988–1.304)	0.081	202.21	<0.001
Model 2	Nutritional status score	0.007(−0.133–0.147)	0.018	0.009	0.926
	Degree of frailty score	1.145 (0.983–1.306)	0.082	193.909	<0.001
	Nutritional status score × Degree of frailty score	0.214 (0.011–0.417)	0.104	4.927	0.039

## 4 Discussion

### 4.1 Analysis of the current situation of sleep quality among older adults

The results of this study showed that 24.3% of older adults self-reported poor sleep quality, which is higher than the findings of Zhang et al. (17) in their sleep quality survey of older adults in Hebei Province, where the incidence of poor sleep quality was 21.0%. A possible reason for this difference might be that a relatively large proportion of the study subjects in this research were aged over 65. However, this result is lower than that of Zhu et al. (18), who surveyed the sleep quality of older adults living in 24 nursing homes and found that the incidence of poor sleep quality was as high as 67.3%. The reason could be that the living environment significantly affects the sleep quality of older adults. In the study, most of the surveyed older adults lived with their families or children, and the proportions of those living alone or in nursing homes were relatively small. As a result, most of them could receive the company and support of their families, meeting their emotional and psychological needs.

Although the incidence rates of poor sleep quality among older adults in China vary across different studies, all results indicate that the incidence of poor sleep quality among older adults in China is relatively high, which is an important factor affecting their quality of life. Poor sleep quality is associated with an increased risk of cardiovascular diseases such as hypertension, heart disease, and stroke. Prolonged sleep deprivation can enhance the body’s stress response, thereby increasing the incidence of cardiovascular diseases (19). Poor sleep quality is also related to insulin resistance, increasing the risk of diabetes in older adults. Poor sleep can lead to poor blood sugar control, which in turn affects overall health (20). In addition, poor sleep quality is closely related to negative emotions such as anxiety and depression. Studies have shown that older adults often experience mood swings due to lack of sleep, increasing their risk of mental health problems (21). In conclusion, poor sleep quality among older adults is an important issue that requires high attention and support from society and families.

### 4.2 Influencing factors of sleep quality among 14,021 older adult individuals

#### 4.2.1 Influence of different demographic characteristics and living patterns on the sleep quality of older adults

The incidence of poor sleep quality among older adult women is 1.463 times that of older adults men, suggesting that older adult women are more susceptible to such issues. This aligns roughly with Li, X et al.’s (22) findings, which indicated that the risk of sleep disorders in older adult women is 1.45 to 1.60 times higher than in men. The decline in estrogen and progesterone levels as older adult women age may cause more frequent nighttime awakenings and difficulty falling asleep. Post-menopausal women, in particular, often experience symptoms like hot flashes and sweating, which can disrupt sleep quality (23). Additionally, older age is associated with poorer sleep quality, as older adults’ sleep structure changes with age, typically showing less deep sleep and more light sleep. These changes can lead to more nighttime awakenings and thus affect overall sleep quality (24). Educational attainment also influences older adults’ sleep quality. The incidence of poor sleep quality among older adults with a junior high school education or higher is 0.745 times that of those with less education. This is consistent with Xue et al. (25) research. Older adult individuals with higher education may have shorter sleep durations, as they might be troubled by excessive thinking, lingering occupational stress, social role changes after retirement, and increased cognitive activities, all of which can impact their sleep. However, some studies (26) suggest that the highly educated older adults generally have better sleep quality, likely because they are more aware of the importance of healthy sleep and take preventive measures. Thus, higher education may enhance cognitive abilities and health awareness, potentially improving sleep, but excessive thinking can also cause stress and anxiety, degrading sleep quality. Studies (27) have shown that rural areas usually have a quieter natural environment, which may contribute to better sleep quality. Noise pollution is one of the most important factors affecting sleep quality, and rural areas usually have lower noise levels, which may provide better sleeping conditions for residents. older adults living alone may feel lonely due to the lack of family company. Loneliness not only harms mental health but also physical health, especially sleep quality, as it can trigger anxiety and depression, major contributors to sleep disorders (28). For those in nursing homes, research findings on sleep quality remain contentious environmental noise, some results are subject to dispute, light, and other residents’ activities can disrupt their sleep (29). Economic status is another factor. The risk of poor sleep quality among older adults with a monthly per capita family income below 2000 yuan is 1.266 times that of those with 2000 yuan or more. older adults in better economic situations are less likely to have declining sleep quality, in line with previous research (30). High-income groups enjoy better living conditions, including access to better healthcare, psychological support, and a comfortable environment, which promote better sleep. In contrast, the economically disadvantaged older adults may face more stress and health issues, leading to poorer sleep quality (31).

#### 4.2.2 Ideal lifestyle and sufficient social support are protective factors for sleep quality

In terms of lifestyle, alcohol intake and smoking can improve sleep structure (32, 33). The reason for this is that some older people may have negative emotions such as anxiety and depression. A small amount of alcohol intake and smoking can improve their psychological state and give them a short period of psychological relaxation, helping them to fall asleep. However, smoking and drinking alcohol to help sleep is not recommended. Because aging reduces alcohol metabolism in older adults, and alcohol may worsen health problems and disrupt sleep (34). Smoking also harms their sleep quality (35), increasing insomnia and nighttime awakenings. Physical exams can detect chronic diseases like hypertension and diabetes in older adults, which affect sleep. Appropriate exercise can regulate the sleep–wake cycle, increase deep sleep time and reduce falling asleep time, enhancing sleep (36). Social activities offer emotional support, reduce anxiety, boost a sense of belonging and lower depression risk, all of which benefit sleep. Social support is vital for older adults’ mental health (37). older adults feeling cared for have more stable emotions, leading to better sleep quality (38).

### 4.3 Correlation between nutritional status, frailty and sleep quality in older adults

In this study, multivariate logistic regression analyses uncovered a complex association between nutritional status and sleep quality among older adults. When analyzed independently (Table 2), malnutrition was significantly correlated with poorer sleep quality (OR = 1.193), which aligns with the findings of Jiang et al. (39) and Lin et al. (40). However, in the fully adjusted model incorporating frailty (Table 3), malnutrition unexpectedly exerted a weak protective effect (OR = 0.856). This directional shift does not represent a biological contradiction but rather indicates statistical confounding: owing to their high covariance, the strong effect of frailty (OR = 5.715) masked the majority of malnutrition’s impact. The biological interplay between nutrition and sleep operates through multiple pathways (41), involving biological mechanisms like hormones, neurotransmitters, etc. (42, 43). Malnutrition can raise cortisol levels (44), disrupt neurotransmitter synthesis [e.g., lack of tryptophan affects serotonin (45), magnesium deficiency impacts GABA synthesis (46)], and a healthy gut microbiota related to good nutrition promotes sleep (47). Thus, while malnutrition independently exacerbates poor sleep, its observed effect is attenuated when frailty is accounted for. This underscores frailty’s dominant role in sleep pathology and suggests that malnutrition’s primary influence on sleep may be mediated through frailty. Comprehensive interventions targeting both nutrition and frailty remain essential to improve older adults’ sleep quality.

Logistic regression results showed that higher degrees of frailty were significantly associated with poor sleep quality. Frailty increase led to declining sleep quality, echoing Xu et al. (48). While poor sleep is linked to frailty, some studies (49) suggest long sleep may protect against it, potentially slowing frailty progression with sufficient sleep time. Research found (50) a significant sleep quality-frailty correlation, especially in women, who are more prone to frailty. Post-menopausal hormonal changes, like reduced estrogen, can disrupt sleep and heighten frailty risk.

#### 4.3.1 Interaction between nutritional status and frailty degree affects older adults’ sleep quality

The generalized linear model reveals that the degree of frailty (*B* = 1.145) has a far stronger independent effect on sleep quality than nutritional status (*B* = 0.007). Although the independent role of nutritional status may be overshadowed by frailty, their interaction term is significant, suggesting malnutrition can impact sleep by worsening frailty. This aligns with prior research. Ni et al. (51) noted that malnutrition speeds up frailty through reduced muscle protein synthesis, weakened immunity, and chronic inflammation induction. In a frail state, energy metabolism disorders and decreased mobility further inhibit sleep-regulating hormone secretion, creating a “malnutrition → frailty → sleep disorders” vicious cycle. Xu et al. (52) also found that the risk of sleep quality decline in older adults with both malnutrition and frailty is 2.1 times higher than those with a single risk factor, highlighting the need for prioritized intervention for this group. To address this, personalized diet plans should be tailored to older adults’ specific needs to ensure adequate protein and energy intake. High-protein diets can improve muscle quality and slow frailty progression, alleviating poor sleep (53). Multidisciplinary teamwork is advisable. Dietitians, doctors, and nurses should form a team to jointly create and execute comprehensive management plans for sleep issues related to older adults’ nutrition and frailty (54). For instance, chronic diseases like diabetes and hypertension, which are common among older adults and can affect nutrition, need active management to avoid negative impacts on nutrient intake (55). Family members should be encouraged to participate in older adults’ diet and exercise plans, offering emotional support and supervision. Regular physical activities such as walking, swimming, yoga, and strength training can boost appetite, enhance muscle strength, and lower frailty risk (56). In daily life, promoting older adults’ participation in interest groups and volunteer activities can enhance their enthusiasm and life participation, improving overall health.

Nevertheless, this study has limitations. Though it reveals the synergistic effects of malnutrition and frailty on sleep quality in older adults, both conditions may be driven by other underlying health problems. For example, uncontrolled chronic diseases (e.g., heart failure, chronic kidney disease, advanced tumors) or acute conditions (e.g., infections, postoperative recovery) can directly cause anorexia, metabolic disorders, and muscle loss, exacerbating malnutrition and frailty. These comorbidities may confound the “nutrition-frailty-sleep” chain. Future studies need to systematically collect acute and chronic disease histories, control for such confounders via stratified analyses or multivariate modeling to validate current findings’ generalizability. Secondly, the nutritional status and degree of frailty of older adults changes over time, so the mechanisms and factors influencing the study are complex, and future research could combine cross-sectional and longitudinal studies to fill the gaps in the field.

## 5 Conclusion

In conclusion, this study focused on older adult population in Zunyi City, aiming to explore the relationships among their nutritional status, degree of frailty, and sleep quality. Our findings revealed that both malnutrition and frailty are significant determinants of poor sleep quality in older adults, and they exhibit an additive interaction effect in this regard. Notably, frailty exhibited a substantially stronger independent association with poor sleep than malnutrition, retaining statistical significance even in highly adjusted models. The attenuation of malnutrition’s effect following frailty adjustment indicates that its influence is largely mediated by the progression of frailty. These findings underscore the critical necessity of prioritizing frailty screening and management—alongside nutritional support—in interventions targeting sleep health improvement among vulnerable older adult population. Such efforts can serve as a valuable reference for designing and implementing interventions that target reducing the incidence of poor sleep quality among older adult individuals suffering from malnutrition and frailty.

## Data Availability

The raw data supporting the conclusions of this article will be made available by the authors, without undue reservation.
