# Supplementary material for: Association of the interaction between nutritional status and frailty level with sleep quality in older adults
Source: Front Public Health. 2025 Sep 23;13:1615643. doi: 10.3389/fpubh.2025.1615643 (PMC12500607; doi:10.3389/fpubh.2025.1615643)
Supplement: Supplementary file 1 [file Table_1.docx]

Supplementary Table 1 : Assignment of Variables

|  | **Assignment** |
| --- | --- |
| Gender | 1 = Male, 2 = Female |
| Educational Level | 1 = Below Junior High School, 2 = Above Junior High School |
| Type of Residence | 1 = Urban, 2 = Rural |
| Living Pattern | 1 = Living with Family Members, 2 = Living Alone, 3 = Living in a Nursing Home |
| Average Monthly Per Capita Family Income | 1 = ≥2000, 2 = <2000 |
| Alcohol Consumption Status | 1 = Non-drinker, 2 = Drinker |
| Smoking Status | 1 = Non-smoker, 2 = Smoker |
| Annual Physical Examination | 1 = Yes, 2 = No |
| Social Activity Participation | 1 = Yes, 2 = No |
| Exercise Habits | 1 = Yes, 2 = No |
| Social Support Status | 1 = Sufficient, 2 = Lack |
| Nutritional Status | 1 = Good Nutritional Status, 2 = At Risk of Malnutrition, 3 = Malnutrition |
| Degree of Frailty | 1 = No Frailty, 2 = Pre-frailty, 3 = Frailty State |
| Sleep Quality | 1 = Good Sleep Quality, 2 = Poor Sleep Quality |
